# 2188. Evaluation of biocide susceptibilities of clinical isolates of *Acinetobacter baumannii*

**DOI:** 10.1093/ofid/ofad500.1810

**Published:** 2023-11-27

**Authors:** Martin M Varghese, Jennifer Cadnum, Piyali Chatterjee, Chetan Jinadatha, Curtis Donskey

**Affiliations:** The Cleveland VA Medical Research and Education Foundation, Cleveland, Ohio; Northeast Ohio VA Medical Center, Cleveland, Ohio; Central Texas Veterans Health Care System, Temple, Texas; Central Texas Veterans Health Care System, Temple, Texas; Cleveland VA Hospital, Cleveland, Ohio

## Abstract

**Background:**

Carbapenem-resistant *Acinetobacter baumannii* (CRAB) is an urgent antimicrobial resistant threat due to its resistance to many antibiotics. There is a concern that some antibiotic-resistant strains of *A. baumannii* might also express resistance to biocides, including disinfectants and antiseptics. However, relatively little information is available on the susceptibility of *A. baumannii* to disinfectants.

**Methods:**

Thirty well-characterized clinical *A. baumannii* isolates were studied, including 24 (%) CRAB isolates and 11 (37%) with qacE1 and amvA efflux pump genes. The efficacy of sodium hypochlorite (1:10 dilution of household bleach) and virex-II 256 (quaternary ammonium disinfectant) was evaluated using a standard disk-based quantitative carrier test method (ASTM E2197-11) in the presence of 5% fetal calf serum. The disinfectants were tested at in-use concentrations with the manufacturer-recommended exposure times. Minimum inhibitory concentrations (MICs) to virex-II 256, povidone-iodine, chlorhexidine, and were determined using a broth microdilution method. Serial passage of *A. baumannii* in subinhibitory concentrations of virex-II 256 were performed to assess the potential for emergence of resistance.

**Results:**

All 30 *A. baumanii* isolates were reduced to undetectable levels (>5.4 log_10_ reduction) by both disinfectants. MICs for povidone-iodine, chlorhexidine, and virex-II 256 were 2343.75 to 9375 µg/ml, 0.62 to 12.5 µg/ml, 1.06 to 2.03µg/ml, respectively. With serial passage in subinhibitory concentrations of virex-II 256, the MICs of virex-II 256 increased 4-fold from 2.03 to 8 µg/ml, and some strains developed increases in chlorhexidine MICS from 12.5 to 50 µg/ml.

**Conclusion:**

Sodium hypochlorite and a quaternary ammonium disinfectant were effective at in-use concentrations against *A. baumannii*, including CRAB strains and isolates with efflux pumps. Exposure to subinhibitory concentrations of quaternary ammonium compounds may result in increases in MICs in these disinfectants and other biocides.

**Disclosures:**

**All Authors**: No reported disclosures

